# A Rare Case of Autoimmune Demyelinating Polyneuropathy and Hydrocephalus Secondary to Pembrolizumab

**DOI:** 10.1177/2324709620916358

**Published:** 2020-04-22

**Authors:** Aakriti Arora, Amar Shere, Kunal Patel, Nuwan Gunawardhana, Ericka LiFuentes

**Affiliations:** 1 Mercer University, Macon, GA, USA; 2 Hackensack University Medical Center, Hackensack, NJ, USA; 3 Florida Atlantic University, Boca Raton, FL, USA

**Keywords:** AIDP, pembrolizumab, immune system, squamous cell carcinoma

## Abstract

Pembrolizumab is a humanized monoclonal antibody that targets the programmed cell death 1 protein (PD-1) receptor and blocks the inhibitory checkpoint interaction between PD-1 and its ligands. This interaction leads to the upregulation of effector T-cells and downregulating regulatory T-cell production. Although this mechanism is essential for the management of cancer, it may lead to decreased self-tolerance with an autoimmune reaction toward healthy functioning tissue. One of the less commonly reported and less understood immune-related adverse events includes neuromuscular complications. We present a rare case of autoimmune demyelinating polyneuropathy and hydrocephalus secondary to pembrolizumab use for cutaneous squamous cell carcinoma of the cheek.

## Introduction

Pembrolizumab (Keytruda) is an immunotherapy agent that directly inhibits the programmed cell death 1 protein (PD-1), which prevents its interaction with program death ligand 1/program death ligand 2 (PDL1/PDL2).^1,2^ By preventing the interaction, T-cells are activated and causes apoptosis of the tumor cells that have PDL1 and PDL2. Pembrolizumab is used for the treatment of advanced melanoma, non–small cell lung cancer, and recurrent or metastatic squamous cell carcinoma of the head and neck.^3^ Immune-related adverse effects with checkpoint inhibitor agents including pembrolizumab are well-documented and can include thyroid dysfunction, colitis, pneumonitis, nephritis, and hepatitis; these are often successfully treated with steroids if recognized early enough.^4^ One such rare neuromuscular complication includes acute inflammatory demyelinating polyneuropathy (AIDP).^4^ AIDP is a variant of Guillain-Barré syndrome (GBS) and arises due to an immunological attack against the myelin sheath of the peripheral nerves and nerve roots.^5^ Although rare, there have been a few case reports demonstrating the development of AIDP secondary to pembrolizumab in the literature. We present a similar case in a patient who developed AIDP secondary to pembrolizumab who also developed hydrocephalus.

## Case Presentation

A 70-year-old Caucasian male with a past medical history of left malar melanoma and prostate cancer was admitted for worsening lower extremity weakness in addition to constipation, urinary retention, and decreased rectal tone. His left malar melanoma was treated with radiation and excision in April 2018, and his prostate cancer was treated with radiation in 2014. In August 2018, he was diagnosed with squamous cell carcinoma of the right malar area. He was treated with Mohs surgery, localized radiation treatment, and 4 out of 5 treatments of pembrolizumab in late 2018. He presented to our medical facility after the fourth cycle of treatment when he slowly began to develop progressive bilateral lower extremity weakness.

**Figure 1.**
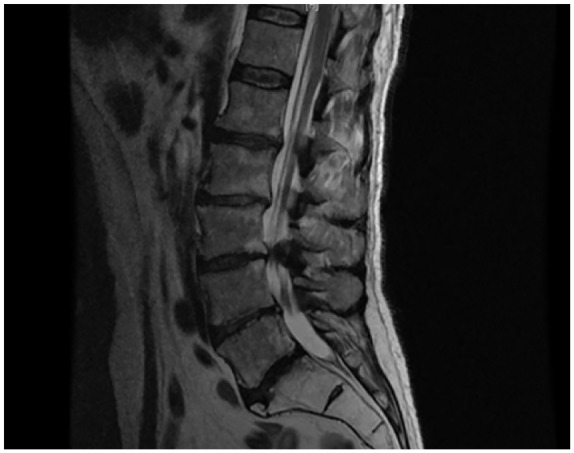
Repeat magnetic resonance imaging results on hospital day 2 revealed abnormal thickened and enhancing posterior nerve roots with L3-L4 to L5-S1 being more severe.

On admission, the patient was afebrile with vital signs as follows: blood pressure 116/73 mm Hg, heart rate 90 beats per minute, oxygen saturation 98%, and respiratory rate 18 breaths per minute. White blood cell count (WBC), complete blood count, and basic metabolic panel did not show any abnormalities. Physical examination was significant for decreased strength in lower extremities (Grades 3-4/5), including the following: mild weakness of right hip flexors, weak bilateral knee flexors, weak left foot dorsiflexion, and plantarflexion. Sensory examination of bilateral feet revealed slight impairment of touch and pinprick sensation. Patellar and ankle reflexes were absent bilaterally. A lumbar spine magnetic resonance image (MRI) revealed abnormal thickening and enhanced posterior nerve roots at L3-L4 and L5-S1 (Figure 1).

Given the clinical presentation and imaging studies, an inflammatory polyneuropathy was suspected. Thus, the patient was started on a 10 mg dexamethasone loading dose and continued on 6 mg every 8 hours. A lumbar puncture (LP) was performed and showed markedly elevated protein at 405 mg/dL and WBC count of 4/mm^3^. On hospital day 6, the patient was started on a 5-day course of intravenous immunoglobulin G (IVIG; 0.4 g/kg). Patient continued to report worsening back pain and lower extremity weakness the following day. Additionally, the patient began to experience painful burning in his feet bilaterally. A repeat LP on hospital day 8 showed cerebrospinal fluid (CSF) protein at 343 mg/dL, WBC at 4/mm^3^, glucose at 41 mg/dL, and negative flow cytometry and cytology that ruled out malignancy. MRI of the brain, cervical, and thoracic spine was performed. Metastatic disease could not be excluded per the thoracic MRI. MRI of the brain and cervical spine showed no features of metastatic disease.

**Figure 2.**
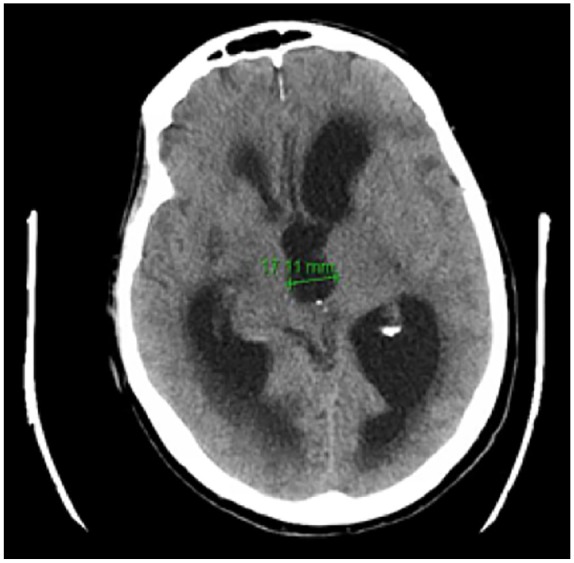
Computed tomography brain scan without contrast revealing internal development of hydrocephalus on readmission.

At this stage, the differential diagnoses pointed toward AIDP likely secondary to pembrolizumab, due to his symptoms, physical examination, abnormal lumbar spine enhancement of the nerve roots on MRI, and an increase in CSF protein. On hospital day 16, the patient was discharged to acute rehabilitation for physical therapy, steroid tapering, and outpatient neurology follow-up with electromyography (EMG) testing. Nine days after discharge, the patient returned to the emergency department from acute rehabilitation for altered mental status. The patient was nonverbal, unresponsive, and bedbound. An emergent computed tomography (CT) brain scan showed internal development of hydrocephalus and an increase in size of ventricles (Figure 2). On physical examination, the patient continued to have lower extremity weakness as was noted in the previous admission. The patient was easily arousable and able to follow commands but confused. He was retreated with dexamethasone for these symptoms.

On hospital day 2 of this visit, the patient continued to complain of leg pain despite therapy. Neurosurgery was consulted and a right external ventricular drain was placed. The cannula was attached to the drainage system at 15 cm H_2_O. The CSF obtained had a negative cytology for malignancy. A repeat CT brain showed unchanged hydrocephalus. On hospital day 3, the patient continued to look extremely ill and CT brain remained unchanged. Per his wife’s wishes the patient was deemed Do Not Resuscitate/Do Not Intubate and eventually transitioned to care and comfort measures only. The patient expired on hospital day 8 after being withdrawn from life support.

## Discussion

Immunotherapy has become increasingly popular among certain cancer treatment options, and as it continues to emerge, rare side effects are being reported. Tumor cells have PDL1 and PDL2 upregulated on the cell membrane. This mechanism allows tumor cells to avoid cell cycle checkpoints and ultimately avoid auto destruction from T-cell lymphocytes. Pembrolizumab binds to PD1 and prevents the interaction between PDL1 and PDL2. By doing so, tumor cells do not pass the cell cycle checkpoint and undergo apoptosis by T-cell lymphocytes. Although this mechanism is beneficial in targeting a primary malignancy, it may result in an autoimmune reaction against nonmalignant tissue (such as peripheral nerves).^4,6^ In some rare cases, pembrolizumab can cause AIDP, a variant of GBS that arises from an immunological reaction against the myelin sheath of the peripheral nerves and nerve roots.^5^

In AIDP, patients experience ascending bilateral limb weakness (more common in the lower extremity), mild sensory loss, hyporeflexia or areflexia, facial nerve paralysis, and dysphagia.^5,7^ The disease course typically lasts an average of 6 weeks, and 5% of patients clinically deteriorate at 8 weeks.^5^ Diagnosis of AIDP is largely based on clinical presentation. MRI often reveals nerve root enhancement within the cauda equina region.^5^ Additionally, CSF studies show elevated protein and albumino-cytologic dissociation in about 50% to 65% of patients. Electrodiagnostic studies (EMG and nerve conduction studies) can be used to confirm the diagnosis of AIDP versus other variants of GBS.^7^

Immune-related adverse neuromuscular effects such as AIDP secondary to PD-1 receptor inhibitors have been rarely documented. A recent single-center retrospective cohort study conducted among 347 patients treated with either pembrolizumab or nivolumab revealed that neuromuscular complications were present in 2.9% of the sample population.^8^ Other studies estimated the incidence to be between 1% and 4.2%.^4,8^ Another case described a patient who was treated with pembrolizumab for stage IV adenocarcinoma of the lung and subsequently developed progressive weakness of the lower extremities.^4^ He was diagnosed with AIDP and was treated with methylprednisolone and IVIG. In another similar case, a patient was treated with pembrolizumab for metastatic melanoma.^4^ After the first month of treatment, the patient complained of progressive weakness in his bilateral lower and upper extremities. LP results indicated albumino-cytologic dissociation, and EMG was consistent with motor and sensory neuropathy.^4^ He was diagnosed with AIDP and was treated with methylprednisolone, IVIG, and plasmapheresis; the patient suffered from a hemorrhage within of his metastatic brain lesions and died soon after.^4^

Moreover, some of the adverse symptoms reported in previous cases include bilateral limb weakness (more commonly involving the lower extremities), paresthesia, hyporeflexia or areflexia, facial nerve paralysis, paresis, ataxia, tremors, and/or dysphagia.^4-6^ Further workup is necessary in these patients and may include imaging of the brain and/or spine with MRI and LP with cytology and flow cytometry. Infectious and paraneoplastic causes should be ruled out. Last, electrodiagnostic studies (nerve conduction studies and EMG) are essential for definitive diagnosis. Treatment of nonambulatory adult patients who present within 4 weeks of neuropathic symptom onset should be started with IV immunoglobulin therapy or plasmapheresis.^9^ In ambulatory adult patients who are not recovering within 4 weeks of neuropathic symptom onset, treatment with plasma exchange and IVIG is recommended.^9^

Our patient was diagnosed with AIDP based on clinical presentation, imaging, and CSF studies. He was treated with high-dose steroids and IVIG with mild clinical improvement as well as decreased CSF protein on repeat LP. He was discharged to an acute rehabilitation facility on high-dose steroids. Unfortunately, he became unresponsive at the facility, was readmitted, and was found to have hydrocephalus; he rapidly deteriorated and died within a week of readmission. Hydrocephalus has not yet been reported as a side effect of pembrolizumab. Therefore, it is important for clinicians to remain highly vigilant for an immune-related adverse event in patients treated with immunotherapy targeted against the PD-1 receptor who present with new neuromuscular symptoms.
